# The Potential of Epigallocatechin Gallate (EGCG) in Targeting Autophagy for Cancer Treatment: A Narrative Review

**DOI:** 10.3390/ijms23116075

**Published:** 2022-05-28

**Authors:** Elena Ferrari, Saverio Bettuzzi, Valeria Naponelli

**Affiliations:** Department of Medicine and Surgery, University of Parma, Via Gramsci 14, 43126 Parma, Italy; saverio.bettuzzi@unipr.it (S.B.); valeria.naponelli@unipr.it (V.N.)

**Keywords:** autophagy, epigallocatechin gallate, cancer therapy, autophagy activator, autophagy modulator

## Abstract

Autophagy is an evolutionarily conserved process for the degradation of redundant or damaged cellular material by means of a lysosome-dependent mechanism, contributing to cell homeostasis and survival. Autophagy plays a multifaceted and context-dependent role in cancer initiation, maintenance, and progression; it has a tumor suppressive role in the absence of disease and is upregulated in cancer cells to meet their elevated metabolic demands. Autophagy represents a promising but challenging target in cancer treatment. Green tea is a widely used beverage with healthy effects on several diseases, including cancer. The bioactive compounds of green tea are mainly catechins, and epigallocatechin-gallate (EGCG) is the most abundant and biologically active among them. In this review, evidence of autophagy modulation and anti-cancer effects induced by EGCG treatment in experimental cancer models is presented. Reviewed articles reveal that EGCG promotes cytotoxic autophagy often through the inactivation of PI3K/Akt/mTOR pathway, resulting in apoptosis induction. EGCG pro-oxidant activity has been postulated to be responsible for its anti-cancer effects. In combination therapy with a chemotherapy drug, EGCG inhibits cell growth and the drug-induced pro-survival autophagy. The selected studies rightly claim EGCG as a valuable agent in cancer chemoprevention.

## 1. Introduction

Autophagy contributes to cellular homeostasis through lysosomal degradation and recycling of redundant or damaged cellular material, which in turn supports cell survival. As well as this basal function, plenty of studies have recognized the role of abnormal autophagy modulation in many human pathological conditions, including neurodegeneration, inflammatory diseases, and cancer, thus suggesting new autophagy-based targets in the rational design of therapeutics [1,2].

In the case of cancer, autophagy plays the role of a double-edged sword, performing a tumor suppressive function, because of its cell protective effects, and a tumor promoting function, by fulfilling the high bioenergetic, biosynthetic, and redox demands of cancer cells at the same time [3,4].

Notably, under chemotherapeutic treatments, autophagy is often upregulated as a cytoprotective mechanism of cancer cells, preventing the damaging effects of anti-cancer drugs [3,5]. Nevertheless, under excessive cellular stress, autophagy may also lead to cell death (type II programmed cell death) and contribute to the regulation of apoptosis (type I programmed cell death) on the basis of a complex molecular crosstalk between the two cell death pathways [6,7]. Likewise, Reactive Oxygen Species (ROS) production is acknowledged to be involved in autophagy induction during cancer therapy, although autophagy may also mitigate ROS production by removing ROS-leaking mitochondria [6].

Based on these paradoxical effects, it is assumed that modulating cellular ROS production and targeting autophagic signaling for selective inhibition, or induction of autophagy, might contribute to sensitizing cancer cells to chemotherapeutic drugs [8,9].

Increasing evidence shows that many fruits, tea, or herbs rich in flavonoids, contain molecules that express anti-cancer properties by regulating cell fate through autophagy and apoptosis [4]. Among them, epigallocatechin gallate (EGCG), from green tea extract, has been in the spotlight for years, and its potential as an antioxidant, anti-inflammatory and anti-cancer agent has been extensively described. In preclinical studies involving different tumor cell lines, a wide range of molecular mechanisms have been ascribed to EGCG, impacting cell proliferation, apoptosis, and autophagy pathways [4,10,11,12]. 

Therefore, in cancer treatment, autophagy represents a promising target that can be effectively achieved by means of the bioactive phytochemical EGCG, via regulating autophagy signaling pathways or autophagy functional status [13,14]. The aim of the present manuscript is to provide an overview of the recent research assessing the anti-cancer effects obtained by targeting autophagy via EGCG treatment in experimental cancer models. In consideration of a comprehensive approach to the topic, we also provide an introductory section concerning EGCG impact on cancer signaling pathways, and we cover details on autophagy pathways in basal and cancerous conditions. 

## 2. Epigallocatechin Gallate: Origin and Application in Cancer Research

The most popular and appreciated herbal teas are produced from the *Camellia sinensis* tea plant. Green, oolong, and black teas are varieties obtained from fresh *C. sinensis* leaves by different manufacturing processes. Green tea preparation requests the sequence of a fixation step (by steaming, pan frying, or sun exposition), a leaf rolling step, and a drying step, whereas oolong and black tea production lacks the fixation step and applies peculiar processes before the final drying step (such as withering and rocking, and withering and fermentation, respectively) [15]. 

Notably, the fixation step is acknowledged to prevent fermentation, ensuring a dry and stable product. In fact, it leads to the inactivation of the enzymes responsible for leaf pigments breaking down and for natural polyphenols oxidizing [16,17,18], thus preserving the highest proportion of bioactive components in the final product as compared with oolong and black teas [19]. 

**Figure 1:**
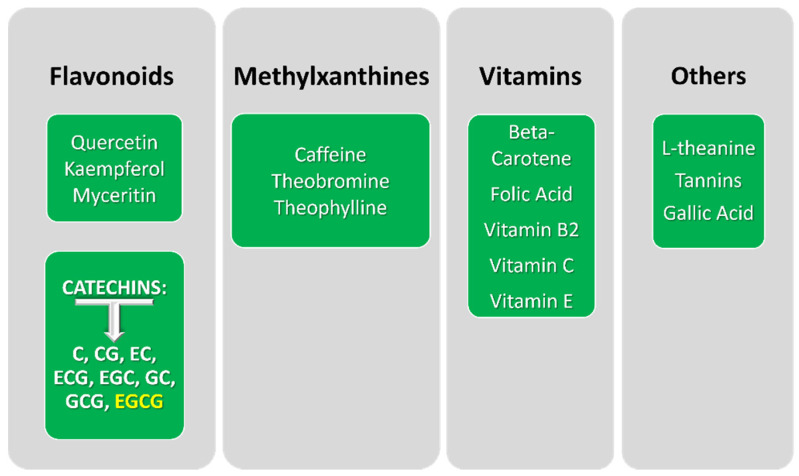
Main categories of bioactive components of green tea. Abbreviations: catechin (C), catechin gallate (CG), epicatechin (EC), epicatechin gallate (ECG), epigallocatechin (EGC), gallocatechin (GC), gallocatechin gallate (GCG), and epigallocatechin gallate (EGCG).

According to Chacko and co-workers, the composition of green tea includes, in order of increasing percentage by dry weight of tea leaves, the following chemical categories: pigments, amino acids, minerals, carbohydrates, lipids, proteins, fiber, and phenolic compounds. Amazingly, the polyphenolic fraction may account for up to 40% of fresh leaf dry mass. Together with methylxanthines, L-theanine, tannins, gallic acid, and vitamins, phenolic compounds constitute a set of bioactive molecules with a potential impact on humans (Figure 1) [17,19]. Among these bioactive compounds, flavonoids represent the most prevalent component, mostly due to the catechins content (Figure 1). 

**Figure 2:**
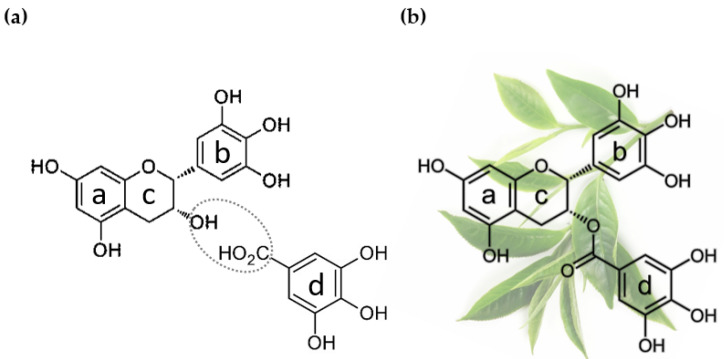
Origin of epigallocatechin gallate. (**a**) Chemical structure of epigallocatechin (EGC, above) and gallic acid (below), the two reactants that form epigallocatechin gallate (EGCG) via esterification of the circled functional groups. (**b**) Chemical structure of epigallocatechin gallate (EGCG), the major constituent of green tea catechins. Background: *C. sinensis* leaves, from which green tea extracts are produced. The two hydroxylated aromatic rings *a* and *b* are connected by a cyclic pyran ring, *c*; the aromatic ring *d* is part of the galloyl moiety, the distinctive element of the gallate derivatives of catechins.

Catechins share the core structure formed by two hydroxylated aromatic rings joined by a pyran ring, as evidenced in the chemical structure of epigallocatechin and epigallocatechin gallate in Figure 2. Green tea catechins comprise the following polyphenolic compounds: catechin (C), catechin gallate (CG), epicatechin (EC), epicatechin gallate (ECG), epigallocatechin (EGC), gallocatechin (GC), gallocatechin gallate (GCG), and epigallocatechin gallate (EGCG) [15,16].

EGCG (Figure 2b) (i.e., the ester of epigallocatechin and gallic acid (Figure 2a)), is definitely the major component of green tea catechins. In fact, (1) it represents 50–80% of the total catechins in green tea [18], and (2) is considered responsible for various health-promoting properties of green tea, after being tested on animals, and subject to cell-based experiments or clinical studies [16]. These health effects range from anti/pro-oxidant, anti-neurodegenerative, anti-microbial, and anti-diabetic activity to anti-cancer activity [13,15,16,17,18,20]. 

Recent developments support the potential role of EGCG in chemoprevention and chemotherapy of various cancers by interfering with cancer initiation, development, and progression. Despite the criticisms often raised in relation to polyphenols stability, solubility, and bioavailability, it has been demonstrated that EGCG can enter the nucleus and interact with DNA and RNA, possibly playing a role in gene regulation [16]. Moreover, EGCG was reported to bind a wide variety of proteins such as kinases, the Epidermal Growth Factor receptor, apoptotic proteins, and the proteasome, thus proving its ability to interfere with multiple signaling pathways [13,16]; therefore, the identification of the molecular targets of EGCG and of the biomarkers that reflect EGCG interactions is essential to deeply understand the potential mechanism of action of EGCG in different experimental set-ups as well as in clinical studies. 

### 2.1. Epigallocatechin Gallate Impact on Cancer-Related Signalling Pathways 

Although numerous health benefits of EGCG have been recognized, the cellular and molecular mechanisms behind them are not yet fully clarified, probably because the action of EGCG impacts multiple cellular pathways, thus simultaneously affecting several processes [21]. Nonetheless, EGCG has emerged as a chemopreventive, and potentially anti-oncogenic product, for the same reason of being capable to target different signaling cascades. In the following subsections, the main signaling pathways known to be influenced by EGCG treatment in different cancer models are briefly described.

#### 2.1.1. ERK and PI3K-Akt Pathways

**Figure 3:**
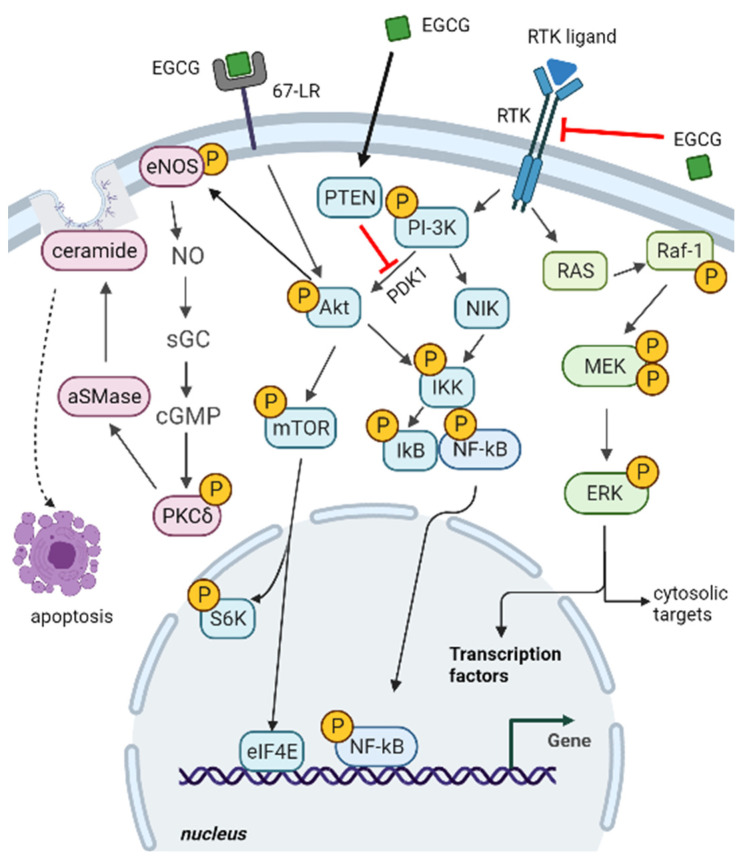
Impact of EGCG on cancer-related signaling pathways. Components of ERK, PI3K-Akt, and 67-LR pathways (from right to left side of the image) are displayed. EGCG interference on ERK and PI3K-Akt pathways may depend on EGCG interaction with a receptor tyrosine kinase (RTK) or PTEN upregulation. EGCG binding to 67-LR may also induce acid sphingomyelinase (αSMase) activation and ceramide generation. See text in 2.1. subsection for a detailed description of the impacted signaling cascades and their implications. Red blunt arrows indicate negative regulation. Black arrows indicate positive regulation. Figure created with BioRender.com (accessed on 20 April 2022).

One of the most investigated pathways modulated by EGCG is the extracellular-signal-regulated kinase (ERK) pathway (i.e., a key signaling cassette of the mitogen-activated protein kinase (MAPK) pathway (Figure 3)). It is triggered by a membrane receptor, such as an activated receptor tyrosine kinase (RTK), and involves a kinase cascade (RAS-Raf-MEK-ERK) to control a large variety of cellular processes. In fact, ERK regulates both cytosolic targets and nuclear transcription factors, thus promoting cell proliferation, differentiation, and survival [22]. ERK activation can also promote oncogenesis and inhibit apoptosis by modulating pro- and anti-apoptotic Bcl-2 proteins [23]. Several studies in cell cultures and animal models demonstrated the inhibition and suppression of this MAPK pathway in response to EGCG administration, mainly through the decrease of ERK1/2 phosphorylation and the inhibition of activity/expression of RAS and Raf-1 [24,25].

The RTK signal also triggers the phosphatidylinositol 3-kinase (PI3K) and Akt/Protein Kinase B (PI3K/Akt) pathway (Figure 3). In response to extracellular signals, PI3K/Akt transduction regulates protein synthesis and cell growth by mTOR complex 1 (mTORC1) activation, and it inhibits apoptosis by blocking the function of pro-apoptotic Bcl-2 proteins. The activity of the kinase mammalian target of rapamycin (mTOR), a core component of mTORC1, turns out to be frequently activated in tumors, and is also a key regulator of autophagy [26,27,28,29]. In fact, over-activation of the PI3K-Akt pathway can result in abnormal cell proliferation, by promoting gene transcription and protein synthesis through the activation of different targets, including eukaryotic translation initiation factor 4E (eIF-4E) and S6 kinase (S6K) [30]. 

Akt and NF-κB inducing kinase (NIK) has a central role in the activation of IkB kinase (IKK) (Figure 3). Once activated, IKK promotes the phosphorylation and the subsequent proteasomal degradation of IkB. The degradation of the inhibitor IkB causes the activation and nuclear translocation of Nuclear Factor kappa-light-chain-enhancer of activated B cells (NF-κB), a small family of inducible transcription factors coordinating cell proliferation during immunity, inflammation, neurodegeneration, and oncogenesis [31]. The chemopreventive action of catechins in the TRAMP mouse model of prostate carcinogenesis is accompanied by clusterin overexpression [32,33]. 

EGCG is acknowledged to interact with RTK receptors that play regulatory roles in cancer signaling [34]. In the case of the epidermal growth factor receptor (EGFR), EGCG inhibits its activation in carcinoma cells, especially in breast cancer and head and neck squamous cell carcinoma, in which activation of EGFR is essential for tumor survival and growth [13]; therefore, the mechanism of the EGCG anti-cancer effect is based on the suppression of the EGFR signaling pathway, involving a decrease of Akt and ERK1/2 activation (Figure 3).

In various cancers, the anti-tumoral effect exerted by the treatment with EGCG has also been evidenced by investigating the phosphatase and tensin homolog (PTEN), a regulator of the PI3K/Akt/mTOR signaling cascade. PTEN prevents the PDK1-mediated phosphorylation of Akt and the consequent mTOR activation (Figure 3). In ovarian and pancreatic cancer cells, EGCG causes an increase of PTEN expression levels and a concomitant decrease of Akt and mTOR activation by phosphorylation, with the resulting suppression of cell proliferation and promotion of apoptotic death [35]. 

#### 2.1.2. 67-LR Pathway

Another investigated target of EGCG is the 67-kDa laminin receptor (67-LR), a cell surface receptor overexpressed in many cancer cells [36,37,38], and drug resistant cancer cells [38]. Many 67-LR receptors lie in lipid rafts (i.e., the dynamic microdomains of the eukaryotic cell membrane that can regulate membrane functions via modulating receptor trafficking and membrane fluidity). Notably, the clustering of lipid rafts in large platforms rich in cholesterol has been associated with aberrant spatial regulation of RTKs, thus playing a role in cancer development and progression [39]. 

A complex signaling pathway involving 67-LR, lipid rafts, protein kinase Cδ (PKCδ), and acid sphingomyelinase (αSMase) has been described in hematological malignancies [40,41]. Briefly, the binding of EGCG to 67-LR may activate Akt, which, in turn, phosphorylates and activates endothelial nitric oxide synthase (eNOS) (i.e., an enzyme that catalyzes nitric oxide (NO) production from L-arginine). The consequence of NO increase is the activation of soluble guanylate cyclase (sGC) with the resulting production of cGMP, which activates PKCδ [42]. Activated PKCδ phosphorylates the αSMase which catalyzes the hydrolysis of sphingomyelin to ceramide (and phosphorylcholine), thus triggering lipid raft-mediated apoptosis [40,41]. Ceramide, in fact, causes the displacement of cholesterol from lipid rafts, determining an increase of membrane fluidity, the formation of lipid raft clusters, and the activation of signaling cascades that culminate with apoptosis. All these events suggest that EGCG, in specific cancer cell types, may modulate lipid rafts-mediated apoptosis via 67-LR, thus expressing its anti-tumor activity. 

#### 2.1.3. Death Receptors-Dependent Apoptosis and Cell Redox Balance

Cancer cells are often characterized by the dysregulation of the apoptotic extrinsic pathway, depending on the activation of cell death receptors (DRs), or of the apoptotic intrinsic pathway, that is mitochondria dependent [43]. Accordingly, many cancer cells present low levels of death receptors which may trigger apoptosis by interacting with the TNF-related apoptosis-inducing ligand (TRAIL). Co-treatment with EGCG and TRAIL, of highly aggressive colon cancer cells, synergistically increased cytotoxicity, by upregulation of death receptors DR5 and activation of caspase 8 [44], demonstrating that EGCG can be a potent TRAIL sensitizer.

EGCG is acknowledged to protect human lens epithelial cells from oxidative stress-induced apoptosis by reducing the generation of ROS and modulating caspase activity [45]; however, although in vitro studies have demonstrated that EGCG exerts a strong antioxidative activity based on its ability to quench free radical species, in vivo studies have proven to be less convincing, since the direct effects on markers of oxidative stress are weaker in comparison with the effects on pathways involved in carcinogenesis [46]. In actuality, because of EGCG moderate/relative oral bioavailability and stability, its tissue distribution and achievement of the effective concentration for direct anti-oxidative effects are non-negligible limiting factors in vivo. 

Instead, data obtained in animal and human subjects highlight the action of EGCG to enhance the expression of detoxifying or antioxidant enzymes such as catalase, superoxide dismutase, glutathione peroxidase or glutathione-S-transferase [47,48]. Presumably, the stimulation of their respective reactions is relevant for the prevention of ROS-induced DNA damage, the enhance of DNA repair machinery, and the inhibition of aberrant cell proliferation [49,50,51]. 

On the other hand, the ability to also induce oxidative stress has been ascribed to green tea polyphenols, associating their pro-oxidant effects to the induction of apoptosis in tumor cell lines [52]. The pro-oxidative potential of EGCG might be ascribed to its autoxidative reactions, resulting in the production of ROS [46]. To date, the combination of EGCG with chemotherapeutic agents represents a protocol used in cancer treatment to increase the oxidative stress in tumors and inhibit the signaling pathways necessary for cancer development and progression [46,53].

## 3. Autophagy Mechanism and Function

In eukaryotic cells, autophagy is defined as the degradation of unnecessary and/or dysfunctional cytoplasmic material by means of a lysosome-dependent mechanism; a regulated self-destructive behavior that maintains energy levels and building blocks for the synthesis of macromolecules and cell homeostasis [3,54]. Although the delivery of waste material to lysosomes may involve different approaches and membrane dynamics, macroautophagy (hereinafter referred to as autophagy) represents the major regulated form of autophagy and is based on the engulfment of a cytoplasm portion by an isolation double-membrane, forming an autophagosome. When the outer autophagosomal and lysosomal membranes merge together, the acidic lysosomal hydrolases degrade the autophagosomal inner membrane and the enclosed material [55,56].

The coordinated action of various proteins drives autophagosome biogenesis. Many of these proteins are coded by evolutionary conserved autophagy-related genes (ATG genes), and together with additional factors, they control the dynamic membrane events involved in autophagy [57,58]. 

**Figure 4:**
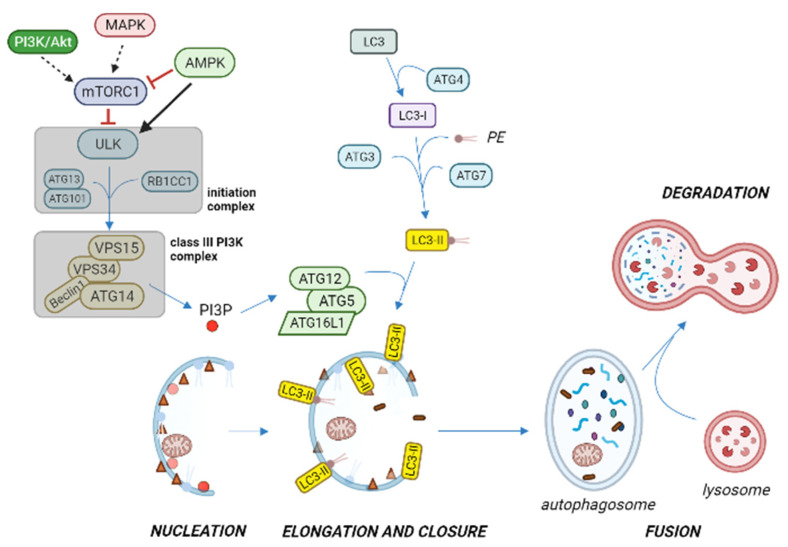
Overview of autophagy activation pathway under stressful or nutrient limiting conditions. The ULK initiation complex induces phagophore nucleation, and translocates to the endoplasmic reticulum or closely related membranes, where it phosphorylates and activates the class III phosphatidylinositol 3-kinase (PI3K) complex, thus producing phosphatidylinositol-3-phosphate (PI3P) on the isolation membrane. PI3P recruits specific autophagy effectors that contribute to ATG12-ATG5-ATG16L1 complex formation, which promotes microtubule-associated protein 1A/1B-light chain 3 (LC3) in conjugation with phosphatidyl-ethanolamine (PE), with the participation of ATG proteins. When the isolation membrane elongates and closes to form the autophagosome, lipidated LC3 (LC3-II) is integrated in the autophagosome, thus becoming a common autophagosome marker. As the autophagosome matures, it fuses with the lysosome to produce the autophagolysosome, where the inner membrane of the autophagic vesicle and its content are degraded by lysosomal hydrolases. Reduced signaling from MAPK and PI3K/Akt pathways on autophagy initiation is represented with dashed arrows. Black arrow and red blunt arrows represent positive and negative regulation, respectively. Figure created with BioRender.com (accessed on 20 April 2022).

The initial steps of autophagosome formation are driven by the autophagosome initiation complex, also known as the ULK complex, that is cytosolic and composed of ULK kinase and its regulatory proteins ATG13, ATG101, and RB1CC1 (Figure 4) [58,59].

Nutrients and energy molecular sensors, such as mammalian targets of rapamycin complex 1 (mTORC1) and AMP-activated protein kinase (AMPK), play a pivotal role in regulating autophagy by acting on the autophagosome initiation complex [55,58,59]. In fact, when in the fed state mTORC1 is activated by amino acids and growth factors (through PI3K signaling), it phosphorylates ULK and ATG13 components of the initiation complex, leading to the inhibition of autophagy. Instead, under stressful or nutrient limiting conditions, mTORC1 is deactivated because of the reduced signaling from phosphoinositide 3-kinase (PI3K/Akt) and mitogen-activated protein kinase (MAPK) pathways, leading to autophagy initiation (Figure 4). In addition, upon energy depletion, AMPK is responsible (by phosphorylation) of the inhibition of mTORC1 and of the activation of ULK complex, resulting in autophagy stimulation [3,54,56,59]. 

In activating conditions, the initiation complex is translocated to the endoplasmic reticulum or closely related membranes, where it induces the autophagosome membrane formation. ULK phosphorylates and activates class III phosphatidylinositol 3-kinase (PI3K) complex (i.e., a protein complex composed of VPS15, VPS34, Beclin 1 and ATG14 proteins), thus triggering the phagophore nucleation. This event results in phosphatidylinositol 3-phosphate (PI3P) signal generation, which marks the precursor membrane for the recruitment of PI3P effector proteins; these, in turn, recruit the ATG12-ATG5-ATG16L1 complex, which promotes the microtubule-associated protein 1A/1B-light chain 3 (LC3) conjugation with phosphatidyl-ethanolamine [54]. Lipidated LC3 (LC3-II) is attached to the phagophore membrane and is required for its elongation and closure. Upon sealing the membrane edges, the autophagosome external membrane acquires SNAp REceptor (SNARE) proteins, which, by interacting with SNAP29 and corresponding lysosomal SNARE proteins, mediate the fusion with the lysosomal membrane, thus starting the degradation phase [56,58].

In physiological conditions, autophagy is induced in response to starvation and aerobic exercise; in these cases, the degradation is intended to produce energetic substrates and amino acids for protein synthesis [58,60,61]. In addition, a basal level of autophagy contributes to the maintenance of cellular homeostasis since it is responsible for the degradation of (1) long lived and misfolded aggregated proteins (protein quality control), (2) dysfunctional mitochondria (mitophagy) and other organelles, (3) membrane lipids and lipid droplets, and (4) various cytoplasmic contents (basal turnover of proteins and nucleic acids). In this sense, autophagy contributes to cell longevity, by promoting cellular quality control, genomic stability, and stem cell maintenance [3,58]. Finally, autophagy also influences immunity and inflammation, because of its potential to eliminate pathogens (xenophagy) and to activate immunity while limiting uncontrolled inflammation [62].

### Autophagy in Cancer

Over the past decade, autophagy has been progressively recognized as a relevant factor in cancer initiation and maintenance, playing a multifaceted and context-dependent role [3,55,56,59].

In absence of disease, autophagy appears to play a tumor suppressive role, given that defective autophagy has been associated with tumorigenesis. In fact, adult mice bearing monoallelic deletion of the Beclin 1 gene showed increased DNA damage and suffered from a high incidence of spontaneous lung and liver cancers and lymphomas [63]. Instead, in human MCF7 breast carcinoma cells, enforced expression of Beclin 1 inhibited the tumor-forming potential of this cell line [64]; therefore, Beclin 1 is an effector of the autophagic pathway (Figure 4), and we may speculate that autophagy is involved in tumorigenesis repression; however, it should be said that the Beclin 1 gene is not specifically mutated or deleted in cancer, but rather, it is lost because of chromosome 17q21 deletions [65]. Nevertheless, the absence of Beclin 1 activators such as the UV radiation resistance-associated gene protein (UVRAG) or Bax-interacting factor 1 (Bif-1) has been associated with colon and gastric cancer development, respectively, reinforcing the idea that autophagy regulation by Beclin 1 might be a key step in some cases of cancer development [56,66,67]. In line with these observations, in early tumorigenesis, a reduced level of autophagy is expected to lead to the accumulation of dysfunctional organelles, reactive oxygen species (ROS), and misfolded/aggregated proteins that may contribute to malignant transformation and cancer progression. 

On the other hand, there is strong evidence that cancer cell growth and maintenance require increased autophagy to meet the metabolic demands associated with high proliferation rates [3,68,69]. For example, acute myeloid leukemia cells are so heavily dependent on autophagy that the inhibition of autophagy was evaluated as a treatment strategy for p53 wild-type acute myeloid leukemia [70]. In this respect, autophagy supports an adaptive metabolic response by providing substrates to almost all carbon metabolic pathways, thus promoting tumor cell survival and possibly resistance to anti-cancer therapies; therefore, it is logical to expect that induction of autophagy might contribute to cancer cell adaptation to hypoxic stress and to reduced nutrient supply, both typical of the unvascularized, metabolically stressed regions of tumors [69,71,72]. 

Finally, autophagy enables tumor cells that lose contact with the extracellular matrix to evade anoikis (a cell death signal that occurs when a cell loses anchorage to the extracellular matrix) and survive, thus favoring cell motility and metastatic spread [3,68]. For example, in hepatocellular carcinoma metastasis, cells rely also on autophagy induction to resist anoikis and spread to distant areas [73]. Nevertheless, by promoting survival in stress conditions, autophagy may also restrict tumor cell necrosis and infiltration of macrophage inflammatory cells at the primary tumor site. Since inflammation constitutes a required step for initiation of the metastatic process, we can assume that autophagy might also attenuate the induction of metastasis [3,56,74]; however, upon intravasation into systemic circulation and colonization of distant sites, autophagy may help the metastatic cells to survive and grow despite the metabolic stresses typical of a new microenvironment [74], thus reflecting, once again, the circumstantial effect of autophagy in cancer.

## 4. Targeting Autophagy in Cancer by EGCG Treatment 

### 4.1. Study Selection

Studies included in this narrative review are papers reporting on autophagy modulation by EGCG treatment in experimental cancer models (cancer cell lines and mouse xenograft). Citations were retrieved by searching PubMed, Scopus, and WoS databases. A flow diagram of the literature search and selection for inclusion in the present narrative review is presented in Supplementary Figure S1. In all the databases considered, the search terms epigallocatechin-3-gallate, autophagy, and cancer were used in association with either treatment, chemoprevention, or other chemotherapy terms. The citation date range considered for study selection was January 2016–September 2021, a period characterized by a considerable interest in EGCG implementation in cancer research (PubMed query: (EGCG) AND (cancer treatment) retrieves more than 500 results). All the included studies are research articles. After full text evaluation, four articles were excluded because of the following features: (1) copy of an included article published on a different journal (N = 1), (2) use of a delivery system of EGCG (N = 2), and (3) autophagy is marginally mentioned (N = 1). For each study, the evidence relevant to this review has been documented in a specifically designed spreadsheet, under the following headers: Cell line/tissue, Principal techniques, Main results, Conclusions, and Reference (Table 1).

### 4.2. Evidence of Autophagy Modulation Combined with Anti-Cancer Effects in Response to EGCG Treatment in Experimental Cancer Models

Among the selected articles (Table 1), ten report an activation of the autophagic flux because of the EGCG treatment. In addition to the direct microscopy observation of autophagosomes, autophagy activation was proven with different approaches, including (1) quantitative analysis of autophagic flux markers (such as LC3, p62, Beclin 1, ATG5) [75,76,77,78,79,80,81,82,83,84]; (2) detection of autophagy pathway-related proteins (such as mTOR, MAPKs, Nrf2, pAkt) [77,79,80,83,84]; (3) visualization of autophagic vacuoles [77,80,81,82,83]; and (4) reversion of EGCG-induced effects by means of genetical or pharmacological inhibition of autophagy (ATG5 shRNA, chloroquine, 3-methyladenine, Bafilomycin A1) [75,76,77,84]. 

Notably, in eight of those studies, EGCG causes proliferation/cell viability inhibition, thus stimulating the apoptotic process [76,77,78,79,80,81,82,84]. Moreover, four of them invoke an excess of ROS generation to rationalize apoptosis and autophagy upregulation [76,77,80,82]. 

In four out of those ten studies, EGCG treatment is associated with a physical stimulation or a pharmacological treatment. In two instances, EGCG is combined with X-ray radiation [79] or low strength pulsed electric field/low energy ultrasound applications [80]. In the other two, EGCG is combined with Tumor necrosis factor-Related Apoptosis inducing Ligand (TRAIL) therapy [75] or all-trans retinoic acid therapy [83].

In the remaining two studies that were reviewed, EGCG treatment is combined with the use of Gefitinib (Gef), an EGFR-tyrosine kinase inhibitor [85], and Doxorubicin [86], a chemotherapeutic drug. In these studies, differently from all the above, cell growth inhibition is accompanied by a reduction of the (pro-survival) autophagy induced by the medication, as suggested by autophagic flux markers expression and vacuoles detection. 

## 5. Discussion

Pharmacologically active plant-derived compounds are gaining interest in the scientific community for their low adverse effects, high availability, favorable cost/effectiveness ratio, and ability to affect different cellular pathways. The most biologically active green tea catechin EGCG has shown marked anti-cancer effects.

Among the different mechanisms that have been investigated to explain the anti-tumoral effect of this catechin, the induction of autophagy-mediated apoptosis is one of the most mentioned [11,41]. According to the included articles investigating the effects of EGCG used alone, EGCG causes autophagy and triggers the process of apoptosis, resulting in cancer cell death without adversely affecting normal cells [76,77,78,81,84]. It follows that there must be a crosstalk between autophagy and apoptosis; although activation of autophagy could, in principle, reduce cell death, in the abovementioned studies, autophagy has a pro-apoptotic role. For instance, according to Wei et al., EGCG upregulates the expression of pro-apoptotic genes, inhibits the expression of anti-apoptotic ones, and induces mitochondrial membrane potential collapse, leading to the activation of downstream caspases and apoptosis [78].

PI3K/Akt/mTOR pathway is targeted by EGCG. Although the molecular mechanisms underlying the combined effect of autophagy and apoptosis have not yet been fully elucidated, a few of the selected studies have examined the signaling pathways modulated by this catechin. EGCG has been shown to induce apoptosis through the PI3K/Akt/mTOR pathway [87]. In this regard, according to Yin et al., both EGCG and the LY294002 inhibitor of PI3K/Akt lead to increased apoptosis in 5637 cell line, whereas their combination synergistically produces the highest efficiency in inducing apoptosis [84]. The Authors demonstrate that EGCG induces PI3K/Akt/mTOR pathway inactivation (required for autophagy induction), resulting in cell growth inhibition by facilitating the crosstalk between autophagy and apoptosis. Correspondingly, Hsieh et al. demonstrate that, in PANC-1 cells, EGCG treatment reduces Akt phosphorylation, suggesting that the downregulation of p-Akt is associated to cytotoxic autophagy induction [80]. These findings are consistent with the literature on PI3K/Akt/mTOR signaling pathway as a “hot spot” target for anti-cancer flavonoids [28].

Pro-oxidant effect of EGCG. Although EGCG has antioxidant as well as pro-oxidant properties, its pro-oxidant activity has been postulated to be primarily responsible for its anti-cancer effects [88]. Tsai et al. observed that, in PEL cells, EGCG induces ROS generation and loss of mitochondrial membrane potential [77]; accordingly, co-treatment with a ROS scavenger reduces the EGCG-induced generation of ROS and cell death, whereas attenuating autophagy and apoptosis. The EGC analogue developed by Xie et al. selectively increases oxidative stress in cancer cells, sparing normal cell lines [76]. Interestingly, their findings also demonstrate that AMPK activation is a link between ROS production and autophagy in cancer cells. Their EGCG derivative, EGCG-palmitate, decreases oxidative stress in normal cells, whereas in cancerous cells it promotes apoptosis and autophagy, uncovering this derivative as a bioprotective antioxidant molecule in normal cells, while showing that it can work as an anti-cancer drug because of its pro-oxidant activity [82]. In this regard, experimental evidence supports the idea that, in comparison to normal cells, cancer cells are more susceptible to hydrogen peroxide, belonging to the endogenous reactive oxygen species, and to hydrogen peroxide-induced cell death [89].

In terms of metabolic implications due to EGCG treatment, Wei et al. focused on the effects of EGCG on glucose metabolism in breast cancer [78]. They demonstrate that EGCG, in addition to its pro-apoptotic and pro-autophagic properties, is effective against cancer development by suppressing glucose uptake, reducing ATP level, and inhibiting glycolytic key enzymes, in opposition to the requirements of growing tumor cells. The study of Zhang et al., although it confirms autophagy and apoptosis involvement in the anti-proliferative process, integrates metabolomic and transcriptomic data to investigate the mechanism underlying the anti-human colon cancer effect of EGCG. Main differential metabolites (induced by EGCG) relate to the pathways of glycerophospholipid and glutathione metabolisms [81], thus reinforcing the hypothesis of a cell redox response.

EGCG in combination therapy. In combination therapy, the effect of EGCG turned out to be amplified by physical stimulation of tumor cells. Administration of EGCG, in combination with a low strength pulsed electric field and a low energy ultrasound treatment, results in increased generation of ROS, autophagy and apoptosis activation, suppression of Akt phosphorylation, and the consequent reduced survival of human pancreatic cancer cells [80]. A reduction of cell proliferation has been also achieved through the administration of EGCG in X-ray irradiated colon cancer cells; the combination treatment induces autophagy, and the apoptotic pathway with the overexpression of caspase 9 [79].

When combination therapy includes EGCG and a chemotherapy drug for cancer treatment, the resulting autophagy status radically changes. In the case of the study by Wang et al., a synergistic effect of EGCG and Doxorubicin on osteosarcoma cell line growth inhibition is observed [86]. The rationale behind this is that EGCG inhibits the Doxorubicin-induced pro-survival autophagy, partly through decreasing SOX2 overlapping transcript variant 7, which is a lncRNA regulator of human cancers. This event reduces the stemness and enhances the chemosensitivity of osteosarcoma cells. Likewise, in the A549 human lung carcinoma cell line, EGCG and Gefitinib synergistically increase the sensitivity to Gef treatment [85]. Moreover, in this case, the EGCG inhibits the autophagic flux induced by the treatment with Gef alone, thus promoting cell death. Finally, the combination treatment partially restored Gef sensitivity through the reduction of ERK and MEK phosphorylation.

Overall, the reviewed studies document a positive trend in bioactive EGCG research field aimed at cancer treatment. Firstly, they demonstrate that autophagy represents a useful target to mitigate cancer cell growth and viability. ROS generation may represent the mean to reach this goal, being able to activate both cytotoxic autophagy and apoptosis. Secondly, combination therapies involving EGCG and non-invasive physical stimulations may synergistically amplify the anti-cancer effects, once again by triggering cytotoxic autophagy. In addition, combination therapy including a chemotherapeutic drug and EGCG is also relevant because of its synergistic effects on cancer cells growth inhibition. The clinical efficacy of conventional chemotherapeutic agents is often compromised by drug resistance; however, the combination therapy with EGCG is able to overcome this chemoresistance via a reduction of the pro-survival autophagy induced by the drug.

The chemopreventive effect of EGCG is dependent on its bioavailability and successful interaction with target tissues; however, EGCG is expected to have low lipophilicity (Figure 2), thus limiting its membrane permeability, especially across the intestinal epithelium [90]. The lack of a receptor-mediated transport suggests that its membrane permeability depends on passive diffusion [91]. 

The therapeutic potential of EGCG in most cases needs a relatively high concentration of catechin. For in vitro studies, an effective concentration of EGCG is usually between 1 and 100 μmol/L; however, this value is difficult to reach in in vivo conditions since the oral administration of green tea catechins results in a plasma peak of tea catechins in the sub- or low-micromolar range [92]. Moreover, the low absorption rate of catechins, their instability in the gastrointestinal tract, and relative bioavailability make it difficult to achieve the therapeutic target. Accordingly, the inconsistency of the biological effects of EGCG obtained in the in vitro and in vivo studies is often due to its poor stability, since under physiological conditions, catechins are rapidly metabolized and transformed in degradation products or in pro-oxidant molecules, regardless of the administration route [93,94]. On the other hand, an excessive amount of catechins may help to achieve proper doses of bioactivity, but it is associated with a dose-dependent toxicological response [95]. Significant efforts are being made to increase EGCG bioavailability and improve its low cellular uptake. Promising results have been obtained with the development of formulations for encapsulating EGCG within hydrophobic nanocarriers [13,16,91,92]. These systems prevent the degradation and metabolization of the transported catechins allowing a higher concentration in the bloodstream [92]. 

Based on EGCG cancer preventive properties, the association between green tea consumption and cancer risk has been investigated in epidemiologic studies [96]. In the prospective study of Kurhashi et al. [97], the authors did not find a significant association between green tea consumption and the risk of being diagnosed with prostate cancer (PCa); however, concerning the risk of development in advanced PCa, a dose-dependent inverse relation was observed when comparing men who consumed five cups of green tea/day with those who consumed one cup/day. 

Moreover, secondary chemoprevention of PCa with green tea catechins produced positive results when studying patients diagnosed with HG-PIN (a premalignant lesion associated with a 30% increased risk of developing PCa within 1 year) and who were treated with catechins [98]. After one year of treatment, only one PCa was diagnosed among the 30 catechins-treated men, whereas nine PCa developed among the 30 placebo-treated men. Clinical trials conducted so far indicate that the window of opportunity for this type of intervention probably corresponds to the early signs of prostate tissue transformation. 

In addition, green tea extract showed preventive effects in patients with oral premalignant leukoplakia, a putative precursor lesion for oral cancer [99].

## 6. Conclusions

The circumstantial role of autophagy, which is reflected in its cytotoxic and pro-survival significance in cancer cells, is confirmed. Its modulation, performed by EGCG administration, was proven to be promising for: (i) developing anti-tumor therapies based on enhancing cancer cell chemosensitivity, (ii) developing anti-cancer strategies based on synergistic effects due to the combination of EGCG and other anticancer drugs, (iii) investigating the nodes of the autophagy signaling pathway, upon which EGCG may impact.

The chemopreventive action of green tea extract is still being debated. Further studies are necessary, not only to improve the stability of catechins but also to produce specific formulations that are able to deliver bioactive EGCG to targeted cells at an effective and safe concentration. These advancements will strongly encourage future in vivo studies and clinical trials which aim to target autophagy for cancer treatment.

## Figures and Tables

**Table 1:** Evidence of autophagy modulation induced by EGCG treatment in experimental cancer models.

Cell line/Animal	Principal Techniques	Main Results	Conclusions	Reference
HCT116 human colon carcinoma cell line	Crystal violet staining for cell viability, LDH cytotoxicity assay, western blotting (LC3, p62), RNA interference	EGCG and TRAIL co-treatment: - increased cell viability; - inhibited TRAIL-induced apoptosis by decreasing the level of death receptors DR4 and DR5; - activated autophagic flux. EGCG effect was reversed by using pharmacological and genetic inhibitors of autophagy (chloroquine and ATG5 siRNA).	*EGCG can protect against TRAIL-induced cell death by activating autophagy in TRAIL-sensitive cells. * *Since autophagy activation may prevent cancer cell death, an autophagy inhibitor is recommended in combination with drugs such as TRAIL because of possible autophagic pathway alterations.*	[75]
B16-F10 mouse melanoma cells, AML-12 mouse hepatocytes, Male C57BL/6 mice	CCK-8 assay for cell viability, flow cytometric analysis of autophagy flux activation (GFP-LC3, Bafilomycin A1 treatment) and apoptosis, flow cytometry with dihydroethidium for measurement of intracellular ROS, B16-F10 xenograft mouse model for in vivo study	In B16 melanoma cells, the EGC analogue 4-(S)-(2,4,6-trimethylthiobenzyl)-epigallocatechin gallate: activated autophagy and reduced cell viability by inducing apoptosis;selectively induced ROS accumulation with consequent cell damage;suppressed tumor growth in vivo, while inducing ROS accumulation.Pharmacological inhibition of ROS by NAC attenuated induced autophagy and apoptosis.	*The EGC analogue has autophagy- and ROS-inducing ability. * *Induced autophagy may act as a downstream sensor of ROS that sequentially induces cell death.*	[76]
Primary effusion lymphoma (PEL) cells (HHV8-positive)	Trypan blue exclusion assay for cell viability, Caspase 3 activity assay, western blotting (LC3, Beclin 1, MAPKs), acridine orange for acidic vesicular organelle staining	EGCG suppressed viral particle production, and inhibited PEL cell line growth. EGCG induced apoptosis and autophagy through ROS generation; 3-MA autophagy inhibitor and Caspase 3 inhibitor failed to rescue the cytotoxic effect of EGCG. NAC co-treatment reduced ROS level, cytotoxicity, Caspase 3 activation, and autophagy in EGCG-treated PEL cells.	*In PEL cells, EGCG induces cell death* via *a mechanism involving ROS generation, leading to autophagy and apoptosis.*	[77]
Mouse 4T1 breast cancer cell line, Balb/c mice	CCK-8 assay for cell viability, flow cytometric analysis of cell apoptosis, Caspase activity assay, western blotting (Beclin 1, ATG5, LC3B), glycolysis-related enzyme activity tests	EGCG inhibited the growth of 4T1 cell line by inducing apoptosis and autophagy. EGCG reduced the expression level of HIF1α and GLUT1, and affected the glycolytic pathway by decreasing activity and/or level of HK, PFK and LDH. EGCG reduced breast cancer xenograft growth in mice.	*EGCG suppresses glucose metabolism and has antitumor activity through the induction of apoptosis and autophagy.* *It might be tested as an adjuvant agent against breast cancer.*	[78]
HCT-116 colon cancer cell line	CCK-8 assay for cell viability, immunofluorescence microscopy (Nfr2 nuclear translocation), qRT-PCR (LC3 and Caspase 9)	EGCG increased cell sensitivity to X-ray irradiation and reduced proliferation.Combination treatment with EGCG and radiation: - increased nuclear translocation of Nrf2 autophagic signal; - induced LC3 and Caspase 9 mRNA expression.	*Combination treatment with EGCG and radiation enhanced the expression of proteins and mRNAs related to autophagy and apoptosis.*	[79]
PANC-1 human pancreatic cancer cell line, HepG2 human hepatocellular carcinoma cell line	MTT proliferation assay, flow cytometry with dihydroethidium for measurement of intracellular ROS, MDC staining for autophagic vacuoles detection, western blotting (LC3, pAkt, Caspase 3 and 9)	Application of low strength pulsed electric field and low energy ultrasound enhanced the growth inhibition effect of EGCG on PANC-1 cells. The triple treatment: - initiated the autophagy pathway through ROS increment; - induced autophagy, that cooperatively caused cell death with apoptosis; - induced cytotoxic autophagy through p-Akt down-regulation and LC3-II upregulation; - overcame the cytotoxicity tolerance of HepG2 cells to EGCG.	*EGCG combined with non-invasive and mild physical stimulations might be a promising strategy for anticancer treatment.*	[80]
HT-29 human colorectal adenocarcinoma cell line	MTT proliferation assay, flow cytometry and TUNEL staining for cell apoptosis, MDC staining for autophagic vacuoles detection, western blotting (LC3B, Beclin1, Caspase 3 and 9), transcriptomics, and metabolomics analyses	EGCG inhibited cell proliferation, and induced apoptosis and autophagy in HT-29 cells. EGCG treatment was associated with significant changes in gene-expression and metabolic profile. Differential metabolites of CRC are involved in the metabolism of glutathione, glycerophospholipids, starch, and sucrose, among others.	*The anti-proliferative activity of EGCG is closely* *related to apoptosis and autophagy.* *Transcriptome and metabolome analyses reveal that the anti-CRC effect of EGCG may depend on its modulation of glycerophospholipids metabolism.*	[81]
HeLa cell line, HEK293 cell line	MTT proliferation assay, DCFDA ROS assay, flow cytometric analysis of cell apoptosis, mRFP-GFP-LC3 plasmid transfection and confocal microscopy, MDC staining for autophagic vacuoles detection, western blotting (LC3, Beclin 1, Caspase 3 and 9)	EGCG-palmitate remained stable in DMEM medium for a longer time than EGCG. In cancerous cells, EGCG-palmitate induced a lower cell proliferation rate, as compared with normal cells, and promoted apoptosis and autophagy, both resulting from excess of ROS generation.	*EGCG-palmitate displayed improved stability and targeted cytotoxicity for cancerous cells.* * EGCG palmitate expresses* *its pro-oxidative bioactivity* *when working as an anticancer drug, and its antioxidant potential in normal cells.*	[82]
HT93, OCI/AML2, MOLM-13 and NB4 human AML cell lines	Western blotting (FASN, LC3B, p-mTOR), shRNA transfection for FASN knockdown, acridine orange for acidic vesicular organelle staining, immunofluorescence microscopy	FASN is upregulated in tumor-associated myeloid cells and becomes a target for autophagic degradation during all-*trans* retinoic acid-induced differentiation of APL cells. Co-treatment with EGCG improved the response to all-*trans* retinoic acid in NB4 cells, and enhanced FASN protein degradation by autophagy. Lowering FASN expression is associated to mTOR pathway inhibition, promoting autophagy.	*Differentiation therapy holds great promise for cancer treatment. * *Co-treatment with EGCG improves the response to all-trans retinoic acid in APL cell lines and significantly re-sensitizes refractory non-APL AML cells.*	[83]
T24 and 5637 human bladder transitional cell carcinoma cell lines	MTT proliferation assay, flow cytometric analysis of cell apoptosis, western blotting (LC3B, Beclin 1, mTOR/p-mTOR, Caspase 3 and 9), shRNA transfection for *ATG5* knockdown	EGCG inhibited proliferation and induced apoptosis in T24 and 5637 cells EGCG regulated apoptosis- and autophagy-related protein expression, and significantly increased autophagosome formation in T24 and 5637 cells. In 5637 cells: - knockdown of *ATG5* reversed EGCG-induced apoptosis; - co-treatment with EGCG and PI3K/AKT inhibitor LY294002 synergically enhanced apoptosis via activation of autophagy.	*EGCG treatment inactivates PI3K/Akt/mTOR pathway, resulting in cancer cell growth inhibition.* *EGCG inhibits bladder cancer cells proliferation by facilitating crosstalk between apoptosis and autophagy.*	[84]
A549 human lung carcinoma cell line, BALB/C male nude mice	MTS proliferation assay, GFP-LC3 plasmid transfection and confocal microscopy, flow cytometry and TUNEL staining for cell apoptosis, western blotting (LC3, ATG5, pERK, p-MEK)	EGCG and Gef synergized in inhibiting the proliferation of Gef-resistant NSCLC cell; the synergy was confirmed also in A549 mouse xenograft models. EGCG inhibited Gef-induced pro-survival autophagy and ERK phosphorylation in A549 cells, thus promoting cell death by apoptosis. EGCG alleviated Gef resistance by inhibiting Raf/MEK/ ERK pathway.	EGCG overcomes A549 Gef resistance by inhibiting Gef-induced autophagy and induced cell death by targeting ERK pathway. This study presents an effective strategy to overcome acquired Gef resistance in NSCLC.	[85]
SaoS2 and U2OS osteosarcoma cell lines	MTT proliferation assay, qRT-PCR (Atg5 and Beclin 1), LC3 immunofluorescence staining, western blotting (LC3), MDC staining for autophagic vacuoles detection, sphere-forming assay	Cell growth inhibition was significantly upregulated when Dox was used in combination with EGCG. EGCG reduced the Dox-induced pro-survival autophagy by decreasing SOX2OT variant 7. EGCG partially inactivated the Notch3/DLL3 signaling cascade, targeting LncRNA SOX2OT variant 7 to reduce the stemness and abate drug-resistance of osteosarcoma cells.	EGCG produced synergistic effects with Dox on osteosarcoma cell growth inhibition by targeting LncRNA SOX2OT variant 7. This study provides a basis for developing anti-tumor treatments targeting osteosarcoma stem cells.	[86]

Abbreviations: TRAIL, Tumor necrosis factor-Related Apoptosis inducing Ligand; NAC, N-acetylcysteine (ROS scavenger); HHV8, Human Herpesvirus 8; 3-MA, 3-methyladenine (autophagy inhibitor); HK, PFK and LDH, hexokinase, pyruvate kinase and lactate dehydrogenase; qRT-PCR, quantitative Real-Time Polymerase Chain Reaction; Nrf2, Nuclear factor-erythroid factor 2-related factor 2; MDC, monodansylcadaverine (marker for autophagic vacuoles); Dox, doxorubicin (chemotherapy drug); NSCLC, non-small cell lung cancer; Gef, gefitinib (EGFR-tyrosine kinase inhibitor); CRC, colorectal cancer; AML, acute myeloid leukemia; APL, acute promyelocytic leukemia; FASN, fatty acid synthase.

## Supplementary Materials

The following supporting information can be downloaded at: https://www.mdpi.com/article/10.3390/ijms23116075/s1. Reference [100] is cited in the supplementary materials.
