# Supplementary material for: The Potential of Epigallocatechin Gallate (EGCG) in Targeting Autophagy for Cancer Treatment: A Narrative Review
Source: Int J Mol Sci. 2022 May 28;23(11):6075. doi: 10.3390/ijms23116075 (PMC9181147; doi:10.3390/ijms23116075)
Supplement: Supplementary file 1 [file ijms-23-06075-s001.zip › ijms-1727163-supplementary.pdf]

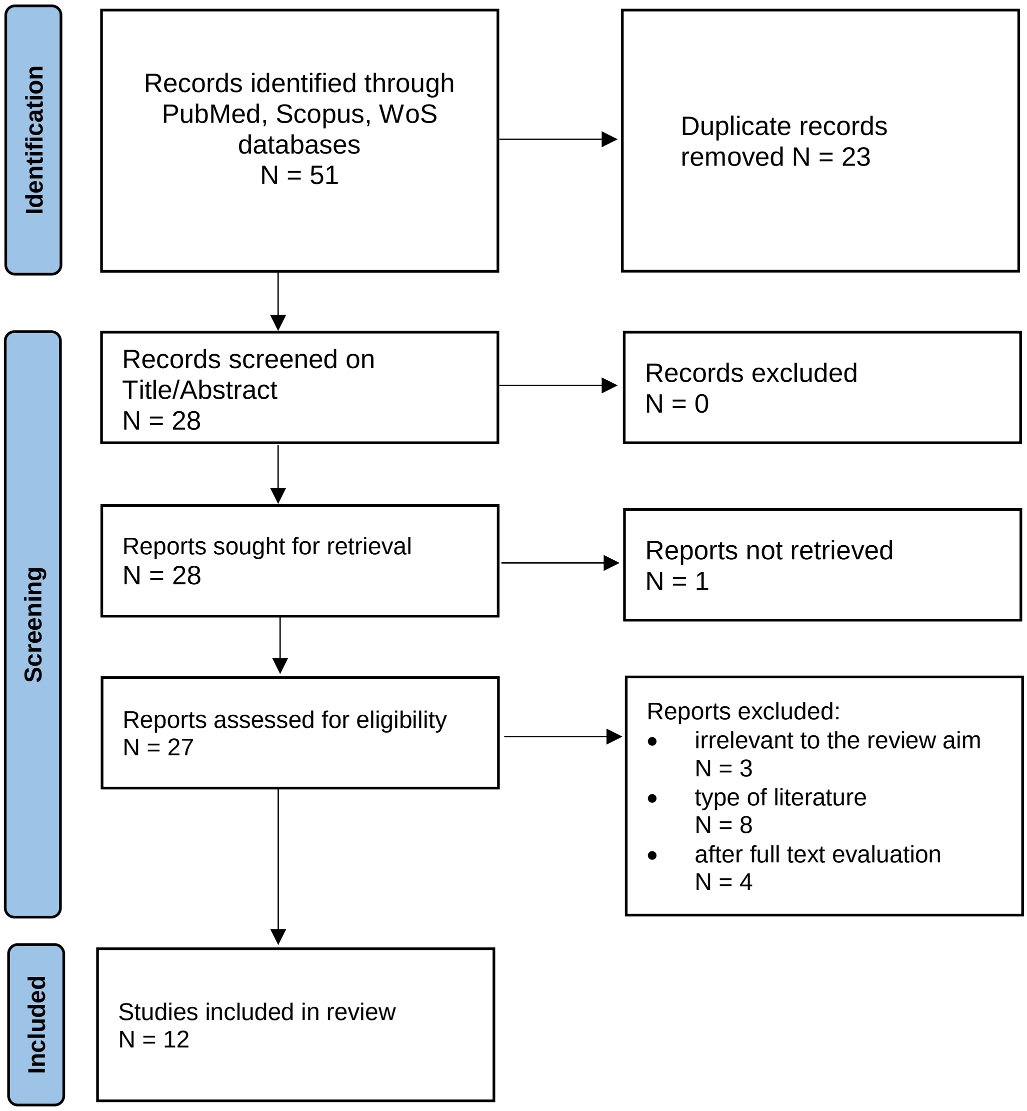

Figure S1. PRISMA flow diagram of literature search and selection for inclusion in the present narrative review (adapted from: The PRISMA 2020 statement: an updated guideline for reporting systematic reviews, BMJ 2021; 372 doi: https://doi.org/10.1136/bmj.n71). All the included studies are research articles. Excluded reports are irrelevant to the aim of the present review (N=3) or are reviews (N=8). After full text evaluation 4 more articles were excluded because of the following features: 1) copy of an included article [91] published on a different journal (N=1), 2) use of a delivery system of EGCG (N=2), 3) autophagy is marginally mentioned (N=1).
